# Thoracic surgery in a hospital dedicated to treating COVID-19: challenges and solutions

**DOI:** 10.6061/clinics/2020/e1982

**Published:** 2020-06-08

**Authors:** Alessandro Wasum Mariani, Paulo M. Pêgo-Fernandes

**Affiliations:** Hospital das Clinicas HCFMUSP, Faculdade de Medicina, Universidade de Sao Paulo, São Paulo, SP, BR

On March 23, 2020, the Central Institute (ICHC) of the Hospital das Clínicas da Faculdade de Medicina da Universidade de São Paulo, was urgently remodeled to treat patients who were suspected or confirmed cases of COVID-19. Seven hundred beds in general wards, and 200 beds in Intensive Care Units (ICU) were quickly made available, with the possibility of adding another 100 ICU beds (1).

The department of thoracic surgery at the Faculdade de Medicina da Universidade de São Paulo, responded with a dedicated team to attend exclusively to the cases in ICHC. This team consisted of three assistants, and two final year and three first year thoracic surgery residents. In addition to attending to all cases of thoracic surgery, the residents also contribute to the regular scale of duties in the ICU. In order to further prevent the spread of COVID-19, it is proposed that the team does not work in other institutes of the Hospital das Clínicas, until the end of the pandemic period.

In preparation, the team members were trained to use personal protective equipment (PPE) (2), as instructed by the Hospital Infection Control Commission. An open channel of direct communication was established between the surgeons and residents of the team.

## Some settings were established:

1. In case of patients undergoing chest drainage that present with pulmonary air fistula, the outlet of air through the drainage bottle could be a source of contamination (3). To mitigate this problem, it was determined that ICU patients with high flow fistulas, should have their drains aspirated (-20cmH2O) routinely. For patients with small fistulas, the drain vent should be equipped with a filter, as described by Barr et al. (4).
2. To meet the demand for tracheostomy requests, a multidisciplinary group was created, comprising of experts from the departments of thoracic surgery, general surgery, and head and neck surgery associated with otorhinolaryngology. A single protocol was agreed by all the members of this group. However, the individualities of each specialist in terms of technique, was respected. One of the concerns was the production of aerosols, therefore, open bedside tracheostomies without the use of an electric scalpel were standardized. In order to minimize the exposure of healthcare workers, it was determined that the surgical team be composed solely of four individuals: one surgeon (staff), one assistant (resident), one intensivist (for sedation and extubation), and one nursing technician. Following the opening of the trachea , a neuromuscular blockade was mandated to prevent cough and aerosol production. Instead of sedation, which is a common practice during bedside tracheostomies, complete anesthesia was also mandated.
3. In addition, the department of thoracic surgery collaborated with other specialties to form Team-ECMO to answer for possible needs of advanced cardio-pulmonary support.
4. Another measure was to initiate a prospective database to better understand the role of a thoracic surgeon in this particular scenario.

After 30 days of activities, the Central Institute have attended to a cumulative number of 1,194 cases. The thoracic surgery team was called to attend to 62 cases. Out of these, 41 patients (66%) had a confirmed diagnosis of COVID-19, and 21 patients were suspected of COVID-19 and were awaiting testing. Nineteen patients (30%) did not need additional procedures. The most common procedures (n=43) included tracheostomy (n=18), and thoracic drainage (n=13). Other procedures were minor interventions, such as tracheostomy tube changes, thoracentesis, and removal of the ‘Montgomery T-tube.’ The only major surgery was a correction of tracheal injury, via endosuture plus tracheostomy. Among all the procedures, we observed only one complication, which was a surgical wound infection following a tracheostomy. However, it was resolved upon following local care measures.

With regards to team safety, PPE were available in 100% of the procedures. However, out of the 43 procedures, a few protocol breaches such as the disconnection of ventilation without an appropriate pause, occurred in six procedures (13%). So far, none of the members of the thoracic surgery/COVID-19 group has displayed signs of the disease.

We conclude that the designation of a specific team of thoracic surgeons, to assist in hospitals dedicated to COVID-19 has produced good results, with regards to the safety of the team and prompt patient service. In the first month, we have verified that the thoracic surgeon's contribution in this scenario is primarily to treat common complications associated with intensive care, and to help chronic cases with the execution of tracheostomy.
